# A maternal diet high in carbohydrates causes bradyarrhythmias and changes in heart rate variability in the offspring sex-dependent in mice

**DOI:** 10.1186/s42826-024-00222-6

**Published:** 2024-09-27

**Authors:** Rosa Elena Arroyo-Carmona, Yareth Mitre-Velasco, Ygnacio Martinez-Laguna, Julián Torres-Jácome, Alondra Albarado-Ibañez

**Affiliations:** 1 Facultad de Ciencias Químicas, Benemérita Universidad Autónoma de Puebla, Benemérita Universidad Autónoma de Puebla, Senda Química, Cd Universitaria, Jardines de San Manuel, Heroica Puebla de Zaragoza, 72570 México; 2 Laboratorio de Fisiopatología Cardiovascular, Centro de Investigaciones de Fisicoquímica de Materiales, Instituto de Ciencias, Benemérita Universidad Autónoma de Puebla 2 Sur 50, San Pedro Zacachimalpa, Heroica Puebla de Zaragoza, 72960 México; 3 Centro de Investigaciones en Ciencias Microbiológicas, Benemérita Universidad Autónoma de Puebla, Heroica Puebla de Zaragoza, México

**Keywords:** Obesity, Arrhythmias, Heart Rate Variability, Offspring, Gender

## Abstract

**Background:**

Maternal obesity prepregnancy, as well as gestational overweight produced by high-sucrose diet, could be evolved to the cardiometabolic diseases in offspring during adulthood. Until then, the cardiometabolic diseases were ignored that have been presented or inherited in the offspring for overnutrition were ignored, depend on gender. We proposed that maternal prepregnancy obesity in CD1 mice, as well as gestational overweight produced by a high sucrose diet, develop to cardiometabolic disease in offspring and even if gender. For detection of the cardiometabolic diseases in a Murine model with a high sucrose diet (HSD), the time series formed by the RR intervals taken from lead I of the ECG has used the corresponding Poincare plot. The heart rate variability was characterized by the standard deviation of width and length SD1, SD2 respectively of the Poincare plot and the SD1/SD2 correlation index in addition was calculated between to gender and body weight.

**Results:**

A maternal diet was based high sucrose diet and produced overweight on progeny in both sexes, but the cardiac arrhythmias depended on gender. Other results were due to the chronic effect of high sucrose diet in offspring with this intrauterine ambiance that contributes to changes in HRV, arrhythmias, and sinus pauses, also these phenomena were observed just in the male mice offspring with high sucrose diet during adulthood.

**Conclusions:**

We propose, that the arrhythmias originated from fetal programming due to the maternal diet in mice model and produced alterations in the offspring female more than in the male, probably due to hormones.

**Supplementary Information:**

The online version contains supplementary material available at 10.1186/s42826-024-00222-6.

## Background

The high consumption of carbohydrates in the diet has been identified as a risk factor for cardiometabolic diseases such as diabetes, insulin resistance, and obesity. Obesity is a complex trait influenced by diet, developmental stage, age, physical activity, and genes. The booming prevalence of obesity is caused by the growing socioeconomic of the country and firstly the use of high carbohydrates increase in the all food industrialization process [1]. Once obesogenic environment factors such as over nutrition, amplify the genetic susceptibilities in all populations and both sexes [2]. However, the global prevalence the obesity in reproductive stage in woman is higher than men, at 20%, 14% respectively [3]. Consequently, childhood obesity could result as a consequence of overnutrition and obesity on the maternal pre-pregnancy, as the obesity had the highest prevalence and numbers in the childhood and play role for prevalence of obesity and increased cardiovascular risk in adults [4], and gestational overweight, these have been associated with non-alcoholic fatty liver disease, dyslipidemia, and insulin resistance in offspring [2, 5]. The imbalanced diets with high carbohydrates during pregnancy, in the offspring could you produce an obesogenic environment and development of an abnormal programming increase the cardiometabolic diseases and reflected in adult age [6]. Also, the rapid increase in obesity from childhood around the globe [7], inevitably overgrowing chronic cardiometabolic diseases such as hyperglycemia, hyperinsulinemia [8], dyslipidemias, hypertension, ectopic adipose tissue [9] and alteration in autonomic nervous system. Age related loss of autonomic nervous system the standard group has been reported as a predisposition factor to the development of the cardiovascular disease [10] and obesity [11], these changes occur dependent on sex [2, 12]. On the other hand, heart rate variability (HRV) has been considered an indicator of general health, also, heart disease, neurological, and metabolism diseases such as diabetes mellitus, metabolic syndrome, and obesity [13–15]. Among the variables that determine the changes in HRV are sympathetic and parasympathetic activity, this last is the activity that predominates in the heart [12]. A method used for quantifying the alterations of the HRV is the Poincare plot because the transversal (SD1) and longitudinal (SD2) parameters of the plot allow measuring the alterations in the electrical activity in the heart due to parasympathetic and sympathetic system [13]. The HRV changes due to obesity in human, in rats [16], and we used it as an early biomarker for diabetes mellitus [6]. This study evaluated the effects on cardiac electrical activity in the offspring (F_1_) of CD1 mice exposed to a high sucrose diet from prepregnancy.

## Methods

### Animal model

**Fig. 1:**
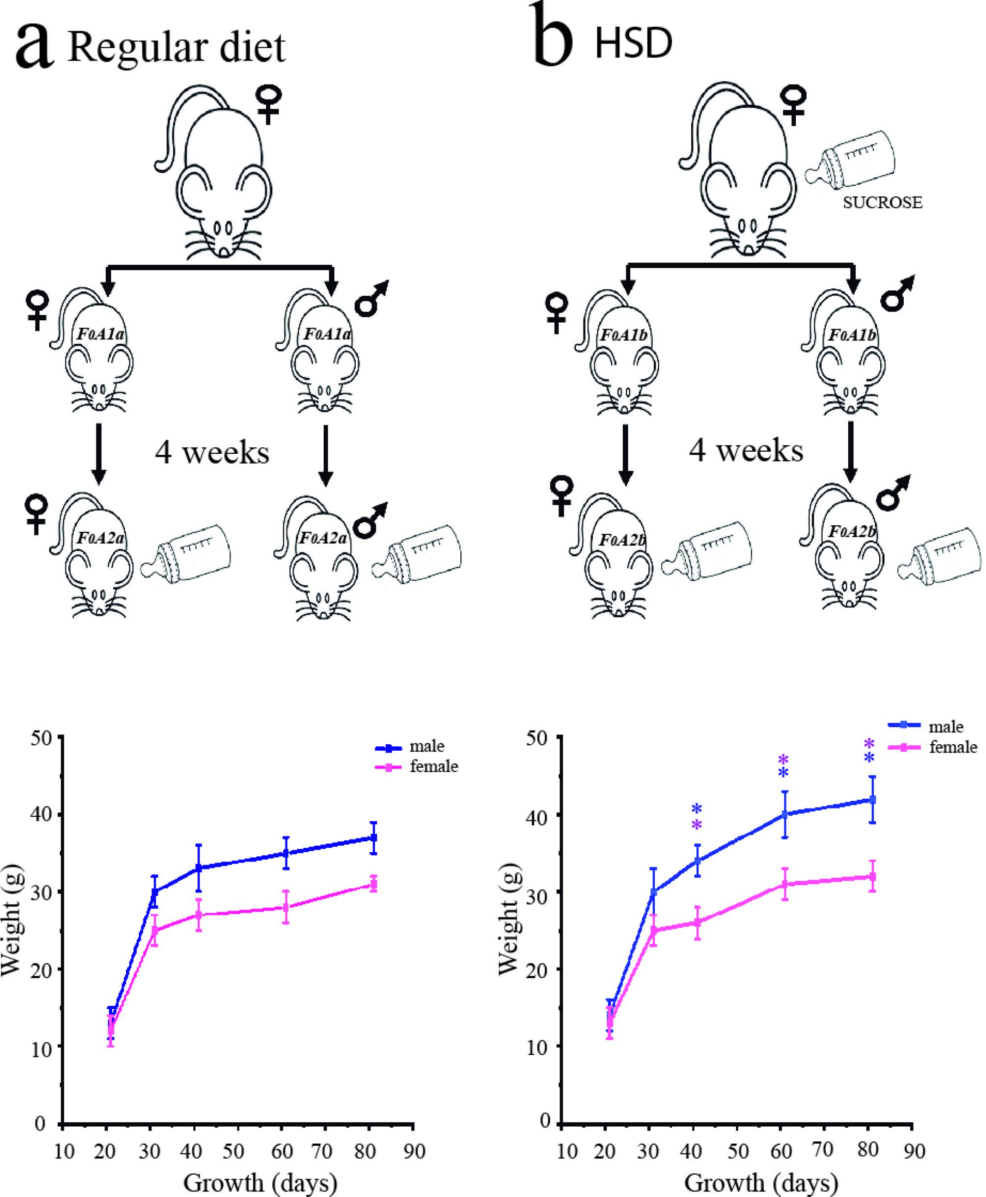
Experimental protocol for studying the effect of maternal diet and the offspring. The regular maternal diet group and their offspring ate regular diet don’t had changes in the growth of the offspring, (**a**). The high-sucrose diet pre-pregnancy in mothers and the offspring had changes in the growth of the offspring, (**b **). Growth in days and weight, lower panel. t-student *p* ≤ 0.05 *female, *male, group at the same age

One group of female mice CD1 strain at eight-week-old was applied to a high sucrose diet (HSD) for four weeks (group b) and the other group was fed with a standard diet (group a). These animals were pregnant, and then were maintained the same treatment during all pregnancy until the weaning of the pups. The pups were on a standard diet (Lab diet 5001) for four weeks (F_1_A1a and F_1_A1b), and at eight-week-old added HSD for four weeks (F_1_A2a and F_1_A2b), (Fig. 1). All animal procedures were performed according to the International Guiding Principles for Biomedical Research Involving Animals Council for the International Organization of Medical Science 2010; including the Animal Care Committee of the Benemérita Universidad Autónoma de Puebla (CICUAL-PROYECTO 00365, 00271).

### Heart rate variability (HRV)

The ECG was performed on the anesthetized mouse (0.5 mg/0.2 mg ketamine-xylazine/kg weight). ECGs were recorded and analyzed using the R-R wave intervals for Poincare plots, and the protocol was described previously by Arroyo-Carmona, et al. [15]. In short, the mice were anesthetized and placed in the supine position, electrodes were placed to record the ECG (using configuration 1), the time interval RR was measured, this interval was called RRi and the time series RRi was made. All points (RRi, RR(i + 1)) were plotted to obtain the Poincaré diagram. In the graph, the distance of each point from the line RRi = RR(i + 1) is measured and the standard deviation of this distance is taken to obtain SD1. To obtain SD2, we draw a perpendicular line to RRi = RR(i + 1) and calculate the distances from each point to this line. Then we calculate the standard deviation, which gives the value of SD2. All data were analyzed using descriptive statistics and expressed as means ± SD (Origin Pro 2017). If the results presented a normal distribution and equal variance, Student’s unpaired t-test. It was assumed to be a significant change if *P* < 0.05.

### Sinus atrial pauses characterization

The sinus pause in the *CD1* mouse model was established with the standard deviation between the RR intervals of ECG, then when the origin the *p* and next wave, exceeded three standard deviations of the RR interval value, it was considered the existence of a sinus pause as an arrhythmia, so for each group of mice was calculated standard deviation of the ECG recorded, supplemental Fig. 1 (S1).

## Results

### The impact of the maternal diet on the weight of the offspring depends on the gender

The effect of maternal HSD in pregnancy produced an increased body weight in the offspring (Fig. 1b) compared with a standard diet (Fig. 1a). After the weaning, both offspring groups had a standard diet over 4 weeks then, the F_1_A1a group had a body weight of 35 g in male and 28 g in female while, the F_1_A1b group had a 40 g in male and 31 g in female, (Table 1; Fig. 1). After 4 weeks of the standard diet, both groups drank HSD for a 4 weeks, (Fig. 1a, b and F_1_A2a and F_1_A2b), of end this diet, these animals showed an increase in body weight to 37 g in male and 31 g in female in the F_1_A2a group, the F_1_A2b group had 43 g in male and 32 g in female. In the maternal diet stage with HSD during pregnancy, the male mice offspring showed a 14% while the female mice presented 11% of overweight, (Fig. 1; Table 1, F_1_A1b group). After the 4 weeks with HSD in adulthood, the female mice F_1_A2a showed overweight at 11% compared with F_1_A2b was 3.2%, and also male mice had overweight of 6% to F_1_A2a and F_1_A2b at 3%. The overweight was dependent on gender, maternal diet, and HSD in adulthood.

**Table 1:** Overweight and heart rate variability of the offspring with maternal exposure to sucrose

	Male				Female			
	F_1_A1a (*n* = 21)	F_1_A1b (*n* = 10)	F_1_A2a (*n* = 21)	F_1_A2b (*n* = 10)	F_1_A1a (*n* = 29)	F_1_A1b (*n* = 12)	F_1_A2a (*n* = 29)	F_1_A2b (*n* = 12)
Body weight (g)	35	40^†^	37 ^†^	43^§^	28^e^	31^‡**∞**^	31^‡**∞**^	32^**∞π**^
Heart rate (BPM)	281 ± 36	274 ± 46	248 ± 51 ^†^	258 ± 46^§^	266 ± 48	264 ± 74	266 ± 49	273 ± 59
SD1	7	9^†^	7	5^§^	9	7^‡^	8	8
SD2	12	14	11	9^§^	16^**∞**^	13^‡^	13	19 ^**π ∞** †^
SD1/SD2	0.7	0.7	0.6	0.6	0.6	0.5	0.9	0.5^†^

Values are means ± SE, t-student significance **†***P* ≤ 0.05 compared with F_1_A1a male; **‡** ≤0.05 compared with F_1_A1a female; **§***P* ≤ 0.05 compared with F_1_A1b male; **π***P* ≤ 0.05compared with F_1_A1b female; **∞** compared with male same age

### The cardiac function of the offspring depended on gender

**Fig. 2:**
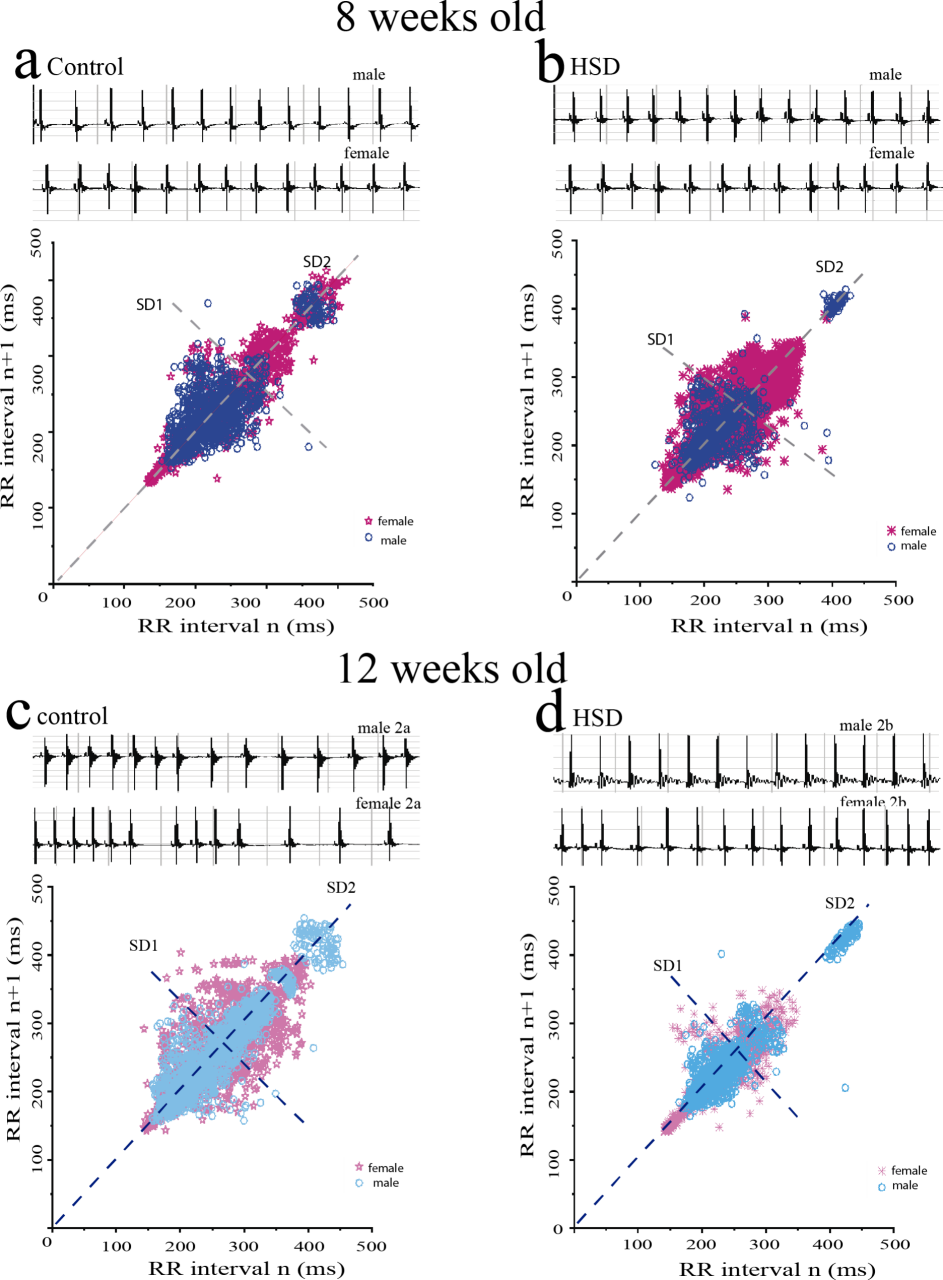
The heart rate variability is dependent on the maternal high-sucrose diet and sex. The offspring of F_1_A1a and F_1_A2a group in male SD1 and SD2 does not change, but female showed an increase in SD2 due to a maternal high sucrose diet, see (**a**, **d**). The offspring of F_1_1b group showed an increase of SD1 and after fed with high-sucrose diet for 4 weeks F_1_2b was observed a decrease in SD1 and SD2 however, the female showed decrease in SD2 by maternal high-sucrose and an increase in SD2 by high sucrose-diet, see (**b**, **c**)

The alterations of the heart rate (HR) in male mice were related to overweight, such as at the exposition with HSD in adulthood both groups showed an increase in body weight and consequence a decrease in HR as reported by Arroyo-Carmona et al., 2016. The F_1_A2a group showed an increase of 6% in body weight and decrease in HR of 12%. The F_1_A2b group reported an increase of 7.5% and a decrease of 6%, (Table 1; Fig. 2). In female mice in the same condition, the heart rate was not related to being overweight.

The F_1_A1b offspring male mice group did not present changes in the heart rate after 4 weeks of weaning with a standard diet, (Table 1). However, in the female mice, the effect of a maternal HSD (F_1_A1b) on cardiac function had consequences in alterations in HRV, the analysis reported changes linked to modulation of the autonomic activity on the heart rate by the parasympathetic system (SD1) and sympathetic (SD2). The HRV showed alterations in the F_1_A1a group in female mice more than in male mice SD1 = 9, SD2 = 16 and, 7, 12, respectively, these alterations are correlated with HSD in adulthood. While, in the F_1_A1b group the female mice reported alterations in HRV less than the female mice F_1_A1a group SD1 = 7 and SD2 = 13, (Table 1; Fig. 2).

**Fig. 3:**
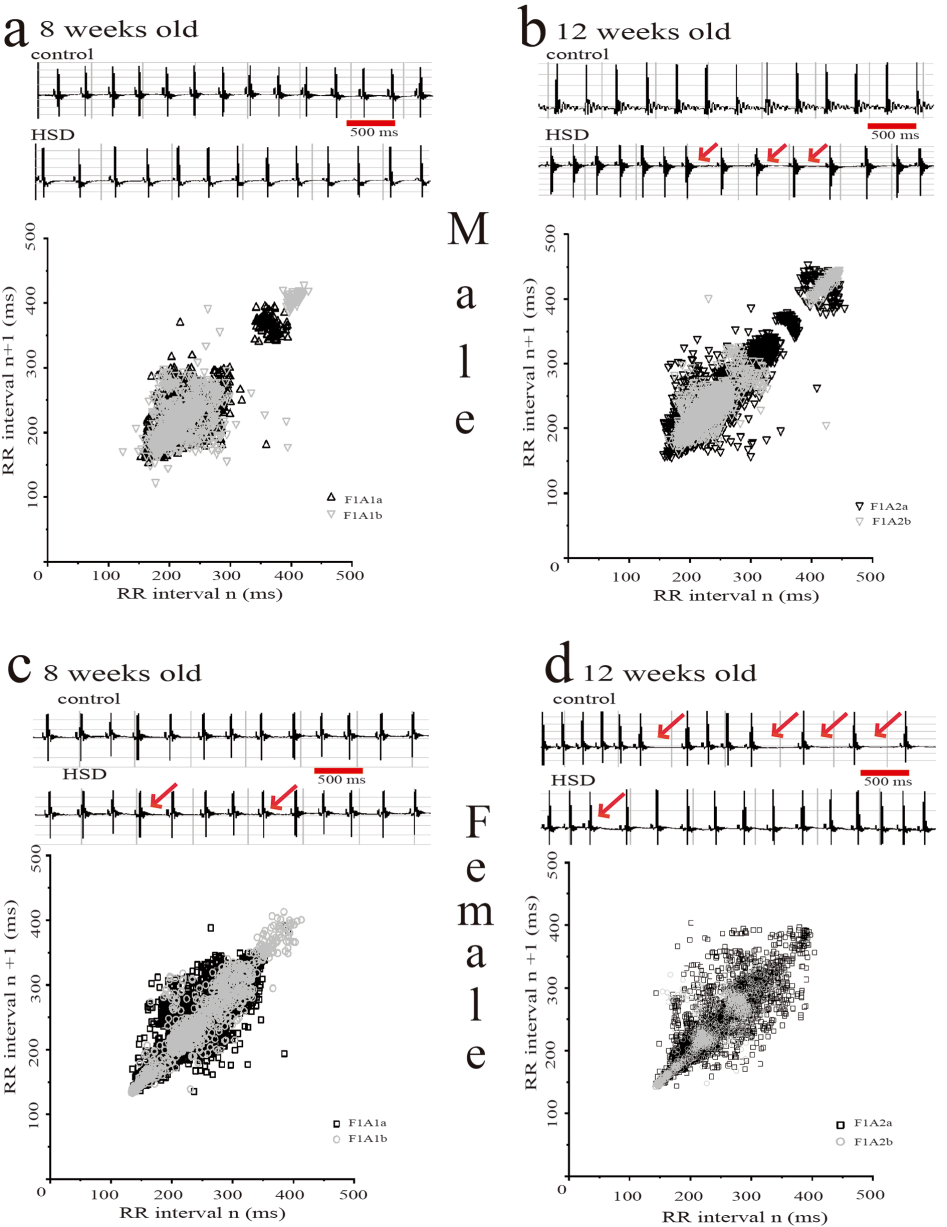
Sinoatrial pause and bradycardia are dependent on the high-sucrose diet and gender. (**a**, **b**) The offspring of F_1_A1b and F_1_A2a, F_1_A2b group in male presented bradycardia, SD1 and SD2 did not change and only F_1_A2b presented sinoatrial pause. (**c**, **d**) But female showed sinoatrial pause in the offspring of F_1_1b and F_1_2a, F_1_2b, and any changes in the heart rate. **Red Arrow showed sinoatrial pause**

However, the HSD for 4 weeks in male mice obtained a loss of 12% in the F_1_A2a group and a decrease of 6% in the F_1_A2b group of change in the HR. In male mice with maternal HSD, the HRV showed an increase in SD2 of 7 for the F_1_A1a group and 9 in the F_1_A1b group. In the same diet condition, in the female mice, the F_1_A1b group, the HRV decreased compared to the F_1_A1a group SD1 value from 9 to 7, and the SD2 value decreased from 16 to 13, respectively. Once the 4 weeks with HSD, the male mice presented alterations in sympathetic and parasympathetic system where the F_1_A2b group had a decreased in SD1 from 7 to 5 and SD2 from 11 to 9 compared to the F_1_A2a group. In the female mice was observed an increased SD2 from 13 to 19 compared to the F_1_A2a, consequently the correlation index SD1/SD2 was 0.5, (Table 1; Fig. 2, and 3). The Poincare plot in female F_1_A2a and F_1_A2b showed a majoF1r variability in both sympathetic as parasympathetic systems only intake of HSD during adulthood.

In the electrical activity heart was present with dangerous arrhythmias, in the first instance in the ECG from F_1_A1b female mice group was observed sinus pause ECG, (Fig. 3). Considering this data, in all mice groups were analyzed the probability of sinus pause, (Figure S1), in the offspring of the mother with a standard diet, the F_1_A1a male mice group, the data showed sinus pause probability of the 9% and after treatment with HSD, the F_1_A2a group, the percentage was zero. In the group of male offspring of mothers with HSD prepregnancy, F_1_A1b group showed 8% and F_1_A2b group at zero of sinus pause probability.

The female mice offspring with regular diet from prepregnancy, the F_1_A1a group presented to 6% probability of sinus pause and the F_1_A2a group probability was 5% of sinus pause (Fig. 3 and S1). However, in the female mice offspring with a HSD from prepregnacy F_1_A1b group did not showa percentage of a probability and F_1_A2b group had a probability percentage of 40% of present a sinus pause, (Fig. 3 and S1).

## Discussion

Many studies have evaluated the effects of maternal high fat or carbohydrate diets during pre-pregnancy on the offspring in animal models and humans [17]. Reporting disturbances in transcriptional and post-transcriptional regulation of genes involved in the metabolism of the obese mother’s offspring [18]. Although fructose exposure during pregnancy has been shown to hyperinsulinemia, impaired insulin signaling, and low adiponectin levels, finally insulin resistance has been reported only in male offspring. Recent reports suggest that the overweight by HSD was associated with decreased heart rate, therefore, cardiac alterations depend on metabolism disorders not only on the weight body [19]. Further metabolic disorders are associated with the rhythm as a decrease in heart rate and dysfunction of the heart (in systole and diastole function) [19], our data showed alterations in rhythm cardiac due to programming fetal maternal by changes in the diet during pregnancy however, the heart rate was not changed as reported in the literature [19]. The heart rate modulation is made by the parasympathetic and the sympathetic system, in the HR remains predominant of the parasympathetic system or SD2 [20]. In addition, we observed that other models had alterations in SD2 or parasympathetic [14]. Thus, we expected that programming fetal was by change in the maternal diet and overweight [6] but in this model the changes in heart rate modulation depended on gender, in male changed the sympathetic system SD1 while in female changed SD1 and SD2 both systems. The SD1/SD2 index in the F_1_A1a group (standard diet) is similar between males and females, however the value of the SD2 is higher in females than in males, hence the balanced in the modulation of heart rate is dependent on gender. In the female mice, the interaction of the parasympathetic system on the heart rate in females is greater than in males consequently the modulation of HRV was imbalanced compared with male, Table 1 [21], suggesting that modulation of HRV is gender dependent, Fig. 3. In the F_1_A1b group, the male offspring have the same SD1/SD2 index as the F_1_A1a group. Although, both groups the intrauterine life produces a change in the interaction of the sympathetic system on heart rate, the above we suppose that F_1_A1b group prevents them from presenting arrhythmias even if they are overweight. Beyond our proposal, the data showed that cardiac remodeling depends on the diet of the mother during the pregnancy and the gender of the offspring. In addition, in later stages of development after giving an HSD to offspring, the remodeling of the HRV was worst in the female offspring. The results showed the F_1_A1b female mice group presented a fetal programming both in the electrical activity of the heart and autonomic nervous system interaction remodeling, (Fig. 2b). The autonomous nervous system acts over reproductive hormones [22], for this reason, we suggest that F_1_A1a offspring females had the SD2 higher than males mice independently of the diet.

The changes in the cardiac electrical activity of ECG reported in this work in both groups exposed to HSD F_1_A2a and b, were similar to metabolic syndrome reported by Albarado et al., 2013. This animal model presented supraventricular arrhythmias with a relationship between overweight and heart rate [19]. Although in F_1_2b male the interaction between the sympathetic and parasympathetic system decreased, while females presented changes in the heart rate variability to SD2 see Fig. 3D, this data suggests that the HRV depends on gender (hormones) and obesity [22].

Found that males of F_1_A2a and F_1_A2b keep the SD1/SD2 ratio unchanged, even though these groups are overweight. In the case of males F_1_A2b, keeps this value constant but in exchange for decreasing SD1 and SD2. In Fig. 3B, the records of the ECG and Poincare diagram of F_1_A2b present a pause of the RR interval without reaching lethal supraventricular arrhythmias. We assume that the intrauterine life promotes the cardiac remodeling of this group to protect it from fatal arrhythmias due to metabolic changes by a diet high in sucrose. The females of F_1_A2a have a value of SD1/SD2 index associated with arrhythmias [14] and it can be seen that the Poincare plot of this group fills a square area, which the data showed an increased fatal ventricular arrhythmia in 9% more than F_1_A2b female, (Fig. 3d). For females of the F_1_A2b group, the SD1/SD2 ratio does not change but increases the value of SD2 increasing the interaction of the parasympathetic with the heart rate variability, this increment is associated with a reduction of the generation of sinus pauses (Fig. 3d, S1), we believe that the maternal diet during pregnancy predisposes her offspring to remain stable in terms of electrical remodeling of the rhythm heart even when the diet changes [23].

## Conclusions

The mouse model with HSD in prepregnancy showed that the offspring male had bradycardia and alterations on HRV. However, the maternal diet combined with high-sucrose-diet in offspring resulted in abnormal HRV related to a nervous autonomous system imbalance depending on sex. In general, the females who changed from a standard diet to a high sucrose diet showed sinusatrial pause, these phenomena are reported in 50% offspring. For this reason, the results of this study support that is necessary to consider the influence of gender differences in cardiometabolic disease research. Also, the model of mice is excellent for researching cardiometabolic alterations in progeny.

## Electronic supplementary material

Below is the link to the electronic supplementary material.

**Supplementary Material 1: Supplementary Figure 1:** Female population showed sinus pause from programming prenatal relationship with high sucrose diet in mother, see the superior panel. The male mice presented sinus pause, although this arrhythmia could be physiological from the maternal environment

## Data Availability

The data used and/or analyzed during the current research are available from the corresponding author on. reasonable request.

## Abbreviations

HSD: High Sucrose Diet
HRV: Heart Rate Variability
ECG: Electrocardiogram
SD1: Standard deviation representative sympathetic system
SD2: Standard deviation representative parasympathetic system
SD1/SD2: Balance between sympathetic and parasympathetic system
F_1_A1a: Offspring first step with standard diet
F_1_A1b: Offspring first step with high sucrose diet
F_1_A2a: Offspring second step with high sucrose diet
F_1_A2b: Offspring second step with high sucrose diet
HR: Heart Rate
