# Supplementary figures and images for: A maternal diet high in carbohydrates causes bradyarrhythmias and changes in heart rate variability in the offspring sex-dependent in mice
Source: Lab Anim Res. 2024 Sep 27;40:34. doi: 10.1186/s42826-024-00222-6 (PMC11428337; doi:10.1186/s42826-024-00222-6)

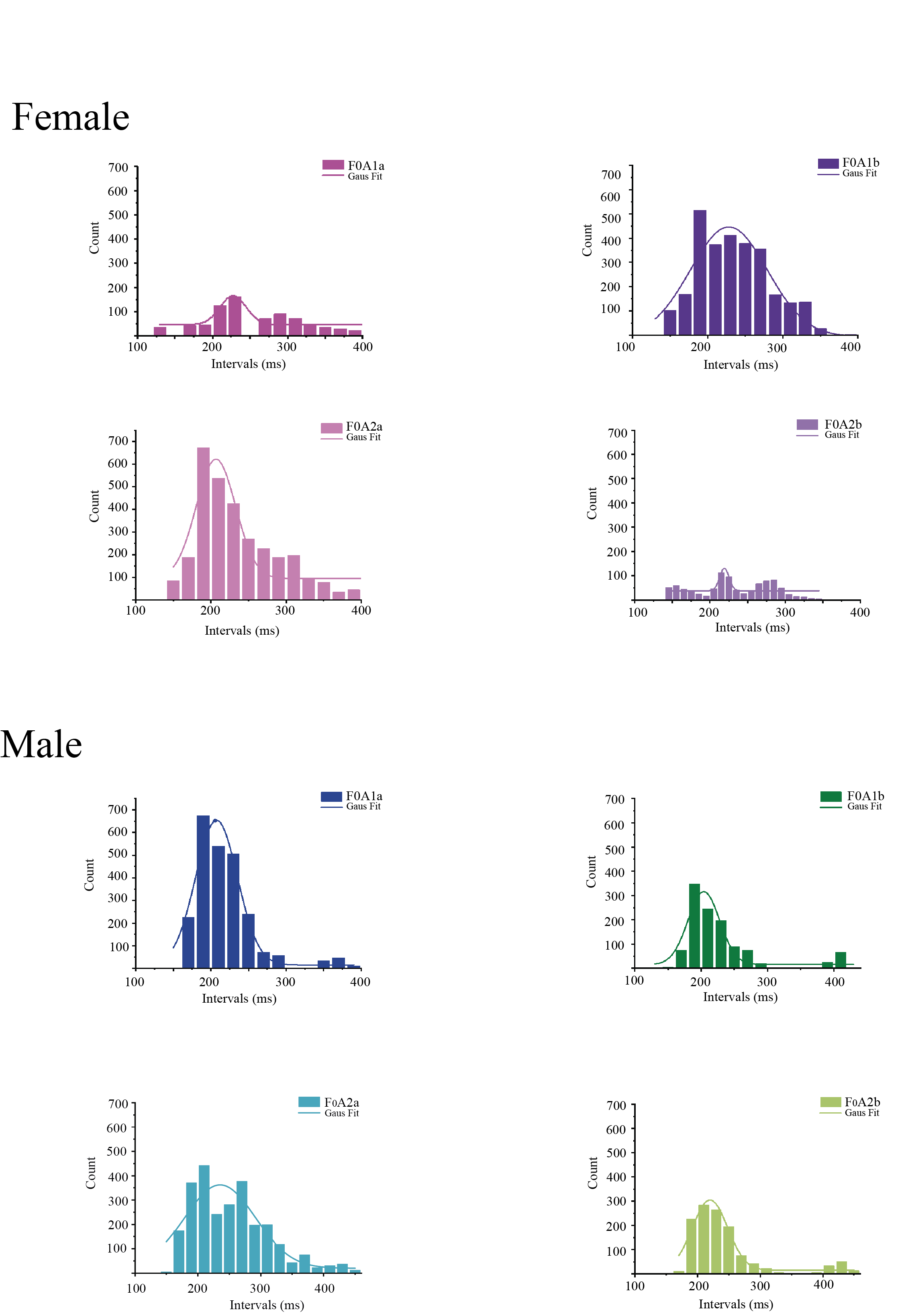

Supplement: Supplementary file 1 — Supplementary Material 1: Supplementary Figure 1: Female population showed sinus pause from programming prenatal relationship with high sucrose diet in mother, see the superior panel. The male mice presented sinus pause, although this arrhythmia could be physiological from the maternal environment [file 42826_2024_222_MOESM1_ESM.tif]
